# Therapeutic Effects of Levocarnitine or Vitamin B Complex and E With Selenium on Glycerin-Treated Holstein Friesian Cows With Clinical Ketosis

**DOI:** 10.3389/fvets.2021.773902

**Published:** 2021-11-12

**Authors:** Seungmin Ha, Seogjin Kang, Manhye Han, Jihwan Lee, Hakjae Chung, Donghyeon Kim, Jinho Park

**Affiliations:** ^1^National Institute of Animal Science, RDA, Cheonan-si, South Korea; ^2^College of Veterinary Medicine, Jeonbuk National University, Iksan-si, South Korea

**Keywords:** therapeutic effect, clinical ketosis, levocarnitine, vitamin B, vitamin E, selenium, glycerin, dairy cattle

## Abstract

Currently, ketosis has no fully satisfactory resolution in dairy cows. Here, we investigated the effect of levocarnitine or vitamin B complex and E with selenium on clinically ketotic cows (β-hydroxybutyrate ≥ 3.0 mmol/L and decreased milk yield), fed glycerin. In total, 18 cases of Holstein cows with clinical ketosis during the postpartum transition period were randomly assigned to three treatments (6 cases per group): (1) levocarnitine (C+G), (2) vitamin B complex and E with selenium (VBES+G), and (3) levocarnitine and vitamin B complex and E with selenium (C+VBES+G). All groups were administered glycerin. Treatments were administered daily for 4 days. Blood sampling was performed on the onset day of ketosis (day 0), day 4, and day 6. β-Hydroxybutyrate (BHBA), milk yield (MY), and serum biochemical values were measured. Half of the animals in C+G failed to overcome clinical ketosis. VBES+G treatment ameliorated BHBA (*p* < 0.05), MY, and glucose on day 4. However, ketosis was exacerbated following the discontinuation of the treatment. C+VBES+G treatment improved BHBA, glucose (*p* < 0.05), and MY and reduced ketotic cases on days 4 and 6 with greater improvements compared to the others. In conclusion, combined treatment with levocarnitine, vitamin B complex and E with selenium, and glycerin may have the therapeutic effect on clinical ketosis.

## Introduction

Ketosis, which is caused by negative energy balance, is a common metabolic disorder during the postpartum transition period in dairy cows. All dairy cattle experience negative energy balance in the early lactation period, which stimulates non-esterified fatty acids (NEFA) release from the adipose tissues to the blood stream (1, 2). NEFA is relatively distributed into one of the three types of metabolism, ATP by complete oxidation, ketone synthesis by incomplete oxidation, triglyceride (TG) production by re-esterification. The incidence of ketosis depends on the extent of lipolysis and tolerance of cows (1, 3).

Ketosis affects global dairy industry. Studies have reported various treatments for ketosis including single or combined use of energy sources (dextrose, propylene glycol, glycerin), pharmacological substances (glucocorticoids, insulin, etc.), and nutritional supplements (amino acids, vitamin B, phosphorus, etc.) (4–15). However, these treatments have ambiguous or side effects and there is limited evidence to support their continued use. Dextrose was not a good treatment option for ketosis, because its effect ends within a day, ketosis recurs, and electrolyte imbalance occurs due to the excretion of surplus glucose via the kidneys (16, 17). Glucocorticoids pose the risk of adverse effects, and their effects are ambiguous; therefore, glucocorticoids are not recommended to treat ketosis. Insulin therapy has limited evidence and is rather expensive. In addition, it is unclear whether phosphorus is necessary for treating ketosis (3).

Single usage of glucogenic precursors (propylene glycol and glycerin) was effective against ketosis, but combination treatments using glucose precursors are more effective than their single usage (18–20). A previous study reported that combination of levocarnitine and methionine with propylene glycol improved ketosis more effectively than propylene glycol alone. However, the study had several limitations and the cases were retreated due to the lack of improvement. Furthermore, the effect of levocarntine and methionine with propylene glycol might not be precise because the treatment product includes amino acids and vitamin B, which influence the ketosis-related metabolism (15).

Glycerin is converted to glucose via the rumen and liver while exerting less toxic effects than propylene glycol (21). Recently, surplus production of glycerin from biodiesel fuel production has reduced its high cost, which makes glycerin an attractive energy ingredient. A previous study reported the effect of combining glycerin with glucagon (22). Glucagon has limited evidence of efficacy and is expensive, similar to insulin. It is still unclear which combination with glycerin is adequate for ketosis treatment.

Amino acids, vitamins, and trace minerals are known to enhance the performance and production of dairy cattle. Levocarnitine is an amino acid with quaternary ammonium, and previously, it was called vitamin B_4_. Carnitine plays vital roles in ameliorating fatty acid metabolism by transporting fatty acids into the mitochondria, supporting the immune system, enhancing the antioxidant system, and improving the reproductive system (23, 24). Vitamin B is synthesized by microflora in the rumen; therefore, mature ruminant animals do not require exogenous vitamin B. However, in dairy cattle with high milk production, studies have demonstrated the beneficial effects of thiamine (25), niacin (26, 27), biotin (28), and folic acid (29). The vitamin B complex participates in metabolism as cofactors or coenzymes, for instance, in the TCA cycle in the mitochondria and the folate, glutathione, and methionine cycles in the cytosol (30). Vitamin E, distributed from the liver to the body after being absorbed and transported from the intestine to the liver, acts as an antioxidant (31, 32). Deficiency of vitamin E and selenium is associated with nutritional myopathy, reduced fertility, retained placenta, metritis, and mastitis, whereas selenium enhances antioxidant properties, immune cells, and embryo survival (33, 34). These supplements improve impaired liver functions.

In this study, we aimed to find an effective combination therapy using glycerin and hypothesized that clinical ketosis could be resolved by stimulating the influx of fatty acids into the mitochondria of hepatic cells (levocarnitine), improving metabolic progress with reducing oxidative stress (vitamin B complex and E with selenium), and supplying a precursor for glucose synthesis (glycerin). Thus, the objective of this study was to determine the effect of levocarnitine or vitamin B complex and E with selenium on the resolution of clinically ketotic cows that were fed glycerin in the context of improved BHBA, glucose, milk yield, and serum biochemical values. The findings could help establish a strategy to overcome or prevent ketosis.

## Materials and Methods

### Animals

The present study was conducted in a farm in the National Institute of Animal Science in Cheonan, Republic of Korea, using a total of 18 cases of Holstein Friesian cows with clinical ketosis. The cows had no other diseases. The cows were raised in a loose housing system and calved from March 2018 to January 2021. The cows were fed total mixed rations *ad libitum* (Table 1). They were milked twice a day, in the morning and evening.

**Table 1:** Ingredients and nutrients of total mixed rations used in this study.

**Item**	**Amount**
**Ingredients composition, % of DM**	
Concentrate	15.32
Soybean meal	2.36
Corn silage	47.13
Alfalfa hay	7.07
Tall fescue	9.43
Timothy	5.89
Energy booster^a^	7.07
Cash gold^a^	4.71
Sodium Bicarbonate	0.12
Limestone	0.16
Zin care^a^	0.12
Supex-F^a^	0.47
Dairyman^b^	0.05
Trace minerals^c^	0.06
Vitamin premix^d^	0.06
**Chemical composition**	
Dry matter (DM), %	53.2
Crude protein, % of DM	12.4
Neutral detergent fiber, % of DM	30.8
Acid detergent fiber, % of DM	18.8
Calcium, % of DM	0.4
Phosphorus, % of DM	0.15

a *Cofavet, Cheonan, Republic of Korea*.

b *Farmtec, Chungju, Republic of Korea*.

c *Contained 0.40% Mg, 0.20% K, 4.00% S, 0.08% Na, 0.03% Cl, 400 mg of Fe/kg, 60,042 mg of Zn/kg, 16,125 mg of Cu/kg, and 42,375 mg of Mn/kg*.

d *Provided ~5,000 KIU of vitamin A/kg, 1,000 KIU of vitamin D/kg, 33,500 mg of vitamin E/kg, and 2,400 mg of vitamin C/kg*.

### Blood Sampling and Study Design

**Figure 1:**
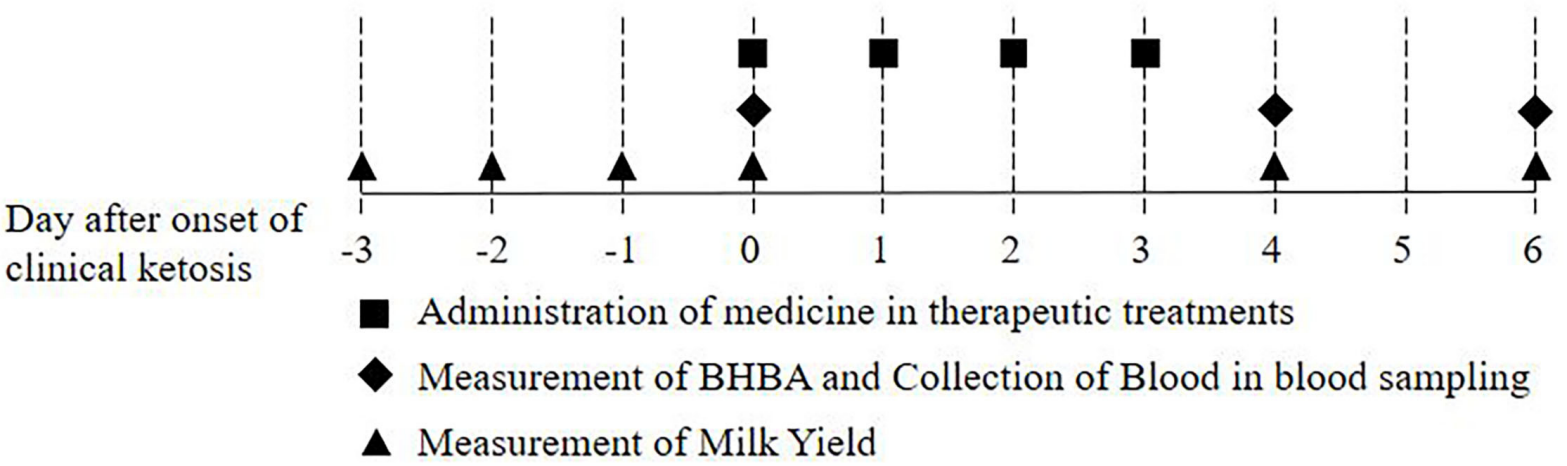
Scheme of this study: Administration of medicine in therapeutic treatments, collection of blood in blood sampling, and measurement of β-hydroxybutyrate (BHBA) and milk yield.

Blood was sampled from the jugular vein of the cows once every 3 days during the postcalving periparturient period (8 times for 21 days from calving date), when the cows began to be fed as soon as they finished milking in the morning. Blood was collected with serum-separating tube (SST) tubes. β-Hydroxybutyrate (BHBA) was measured using electronic handheld meters (FreeStyle Optimum Neo, Abbott Diabetes Care Ltd., Witney, UK) and β-ketone test strips (FreeStyle Optimum H β-ketone, Abbott Diabetes Care Ltd., Witney, UK) immediately after blood sampling. The cows were divided into three groups according to BHBA concentration: non-ketotic (<1.2 mmol/L), subclinically ketotic (1.2 ≤ BHBA ≤ 2.9 mmol/L), clinically ketotic (≥3.0 mmol/L) (35, 36). The clinically ketotic cows used in this study decreased milk yield (MY) over 10%, compared to the previous day for the last 2 days before beginning treatment. Cows with clinical ketosis were randomly assigned to one of three treatment groups (6 cases per group): (1) levocarnitine (C+G) (Carnitine Plus Inj., Unibiotech Co., Ltd., Anyang, Korea), (2) vitamin B complex and E with selenium (VBES+G) (Vitacom Inj., Handong Co., Ltd., Seoul, Korea, and Selevit, Fatro, Ozzano dell'Emilia, Italy), and (3) levocanitine and vitamin B complex and E with selenium (C+VBES+G). Glycerin was administered orally to all groups consecutively for 4 days (500 ml, Eloglyn 995D, LG Household & Health Care LTD., Seoul, Korea), based on a previous study (37). Levocarnitine was administered intravenously 10 g at the first treatment and 3 g for 3 days thereafter. Regarding vitamin B complex, thiamine hydrochloride 250 mg, riboflavin sodium phosphate 10 mg, pyridoxine hydrochloride 10 mg, and nicotinamide 250 mg were injected intravenously for 4 days, whereas cyanocobalamin 1 mg was administered subcutaneously. Vitamin E (α-tocopherol, 700 mg) and selenium (1.5 mg) was administered subcutaneously for 4 days. The treatments were administered, following the administration method and dosage of each medicine. Blood sampling was conducted 1 day (day 4) and 3 days (day 6) after the final treatment in the morning (Figure 1).

### Blood Analysis and Data Collection

Serum was harvested by centrifuging SST tubes at 3,000 rpm (2,600g) for 10 min. The serum was frozen and stored at −70°C pending analysis. The serum biochemical analysis was performed using a biochemistry automatic analyzer (Hitachi 7180, Hitachi Ltd., Tokyo, Japan). The levels of glucose, total protein (TP), albumin, NEFA, TG, alanine transaminase (ALT), aspartate transaminase (AST), and gamma-glutamyl transferase (GGT) were measured by commercial enzyme assay kits made by Wako (Fujifilm Wako Pure Chemical Ltd., Osaka, Japan). Globulin levels were calculated as the differences between TP and albumin values. Total bilirubin (TB) was analyzed immediately after centrifugation by using Catalyst™ Dx chemistry analyzer. MY was obtained through a milking metering system (Alpro system, DeLaval, Tumba, Sweden). Change of MY was obtained by subtracting milk yield on the onset of ketosis from milk yield on specific date, and dividing that by milk yield on the onset, and then multiplying that with 100 [(MY_(Dayx)_ - MY_(Day0)_)/MY_(Day0)_ * 100].

### Statistical Analyses

Statistical analyses were performed using SPSS software (version 26.0; IBM Corp., Armonk, NY, USA). Repeated-measures analysis of variance was used to assess the levels of BHBA, glucose, TP, albumin, NEFA, TG, ALT, AST, GGT, and milk yield on days 0, 4, and 6. Time and treatment were the fixed effects, while cow nested within the group was the random effect. Group and time were the main effects, and group-by-time was the interaction effect. When the effects were significant (*p* < 0.05), the analysis of variance with *post hoc* Bonferroni correction was used to evaluate the difference by time or group. Paired *t*-test was used to compare within-group categorical variable changes by time. All data are expressed as the mean ± standard deviation (SD). Significance level was interpreted based on the *P*-value; 0.01 ≤ *p* < 0.05 significant, 0.001 ≤ *p* < 0.01 very significant, and *p* < 0.001 extremely significant.

## Results

### Descriptive Statistics for Dairy Cows by Each Treatment

In total, 18 cases of Holstein Friesian cows with clinical ketosis were analyzed according to the treatment type. The levocarnitine (C+G) group calved less and was younger than the other groups. The average incidence days of clinical ketosis were the highest in the C+G group. The levocarnitine and vitamin B complex and vitamin E with Selenium (C+VBES+G) group had the oldest cows and highest parity, and they suffered from clinical ketosis earlier than cows from any other group (Table 2).

**Table 2:** Descriptive statistics for dairy cows by each treatment included in the study.

**Variable**	**C + G**	**VBES + G**	**C + VBES + G**	***p*** **-value**
Number	6	6	6	
Cow parity	2.33 ± 1.21	3.00 ± 1.55	3.00 ± 2.10	0.729
Age at calving	4.77 ± 1.31	6.48 ± 2.12	6.62 ± 3.16	0.333
Body condition score	3.17 ± 0.20	3.04 ± 0.33	3.15 ± 0.45	0.790
Incidence day of clinical ketosis	16.50 ± 4.14	10.50 ± 4.14	9.50 ± 4.42	0.024

*Data are presented as the mean ± standard deviation. C+G = levocarnitine and glycerin, VBES+G = vitamin B complex, vitamin E with selenium, and glycerin, C+VBES+G = levocarnitine, vitamin B complex, vitamin E with selenium, and glycerin*.

### Changes in β-hydroxybutyrate and Glucose Concentration by the Treatments

**Figure 2:**
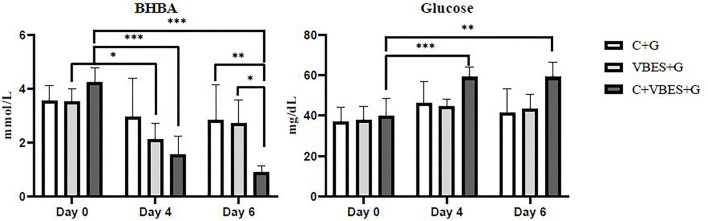
BHBA and glucose concentrations by group and time. The BHBA and glucose concentration were measured on the pre-treatment day (Day 0), and on 1 day (Day 4) and 3 days (Day 6) after the last treatment day. C+G = levocarnitine and glycerin, VBES+G = vitamin B complex, vitamin E with selenium, and glycerin, C+VBES+G = levocarnitine, vitamin B complex, vitamin E with selenium, and glycerin. Data are indicated as the mean ± standard deviation. ^***^p < 0.001; ^**^p < 0.01; ^*^p < 0.05.

The effects of 3 kinds of treatments on BHBA and glucose of Holstein Friesian cows with clinical ketosis were investigated. The tests of between-subjects effect were very significant in glucose (*p* = 0.006), but not significant in BHBA (*p* = 0.081). In the tests of Within-Subjects Contrasts, the main effects of time were extremely significant in both BHBA and glucose concentrations (*p* < 0.001). The interaction effect of group-by-time was extremely significant in BHBA (*p* < 0.001), but not significant in glucose (*p* = 0.069). The difference of BHBA between groups was not significant on day 4 (the day after the last treatment), but on day 6 (3 days after the last treatment). Half of C + G group failed to overcome clinical ketosis on both day 4 and 6. VBES + G was ameliorated on day 4, but BHBA of this group increased on day 6 and a third of the group returned to clinical ketosis on day 6. All cases of C + VBES + G group overcame clinical ketosis on both day 4 and day 6. The decreased amount of BHBA was the biggest among groups. Most cases of C + VBES + G group revived to non-ketosis on day 6 (83.3%), while one case slightly failed to overcome subclinical ketosis (1.2 mmol/L). C + VBES + G group significantly improved glucose concentration after the treatment, while the other groups made little improvements on both day 4 and 6 (Tables 3, 4 and Figure 2).

**Table 3:** Interaction effect of time and group on BHBA and glucose concentration by repeated measures ANOVA.

**Source of variation**	**Sum of squares**	**Degrees of freedom**	**Mean squares**	***F***	***p*** **-value**
**BHBA**					
Time	30.535	2	15.267	34.464	<0.001
Time*Group	14.775	4	3.694	8.338	<0.001
Error	13.290	30	0.443		
Group	7.194	2	3.597	2.989	0.081
Error	18.052	15	1.203		
**Glucose**					
Time	1,408.037	2	704.019	14.759	<0.001
Time*Group	463.630	4	115.907	2.430	0.069
Error	1,431.000	30	47.700		
Group	1,439.148	2	719.574	7.434	0.006
Error	1,451.833	15	96.789		

**Table 4:** Percentage of ketotic types on pre-treatment day (Day 0), and on 1 day (Day 4) and 3 days (Day 6) after the last treatment day.

	**C** **+** **G (*****n*** **=** **6)**			**VBES** **+** **G (*****n*** **=** **6)**			**C** **+** **VBES** **+** **G (*****n*** **=** **6)**		
	**NK**	**SCK**	**CK**	**NK**	**SCK**	**CK**	**NK**	**SCK**	**CK**
Day 0			100			100			100
Day 4		50	50		100		50	50	
Day 6	16.7	33.3	50		66.7	33.3	83.3	16.7	

*NK, non-ketosis; SCK, subclinical ketosis; CK, clinical ketosis. C+G, levocarnitine and glycerin; VBES+G, vitamin B complex, vitamin E with selenium, and glycerin; C+VBES+G, levocarnitine, vitamin B complex, vitamin E with selenium, and glycerin*.

### Changes in Daily Milk Yield by Treatments

**Figure 3:**
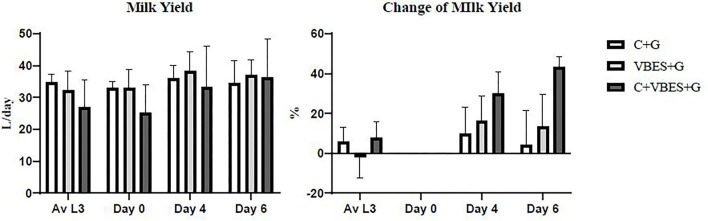
Daily milk yield and change in daily milk yield compared with the onset day of ketosis. The value of Av L3 indicates the average of daily milk yields for the last 3 days before the onset day of ketosis. The values on days 0, 4, and 6 indicates daily milk yields on the pre-treatment day, 1 day after the last treatment day, and 3 days after the last treatment day, respectively. C+G = levocarnitine and glycerin, VBES+G = vitamin B complex, vitamin E with selenium, and glycerin, C+VBES+G = levocarnitine, vitamin B complex, vitamin E with selenium, and glycerin. Data are presented as the mean ± standard deviation.

Improvement in MY was measured depending on the type of treatment. MY was not statistically significant. All groups showed increased daily MY on day 4 compared with the average MY during the last 3 days before ketosis onset and that on day 0. The C+VBES+G group showed a greater increase in MY compared with the other groups from day 0 to 4. The C+VBES+G group showed an increase in MY from days 4 to 6. The C+G and VBES+G groups showed decreased MY on day 6 compared with that on day 4. Although the gap in MY among the groups on day 6 was small, the change in MY in the C+VBES+G group was the largest (Figure 3).

### Changes in Serum Biochemical Values by Treatments

**Figure 4:**
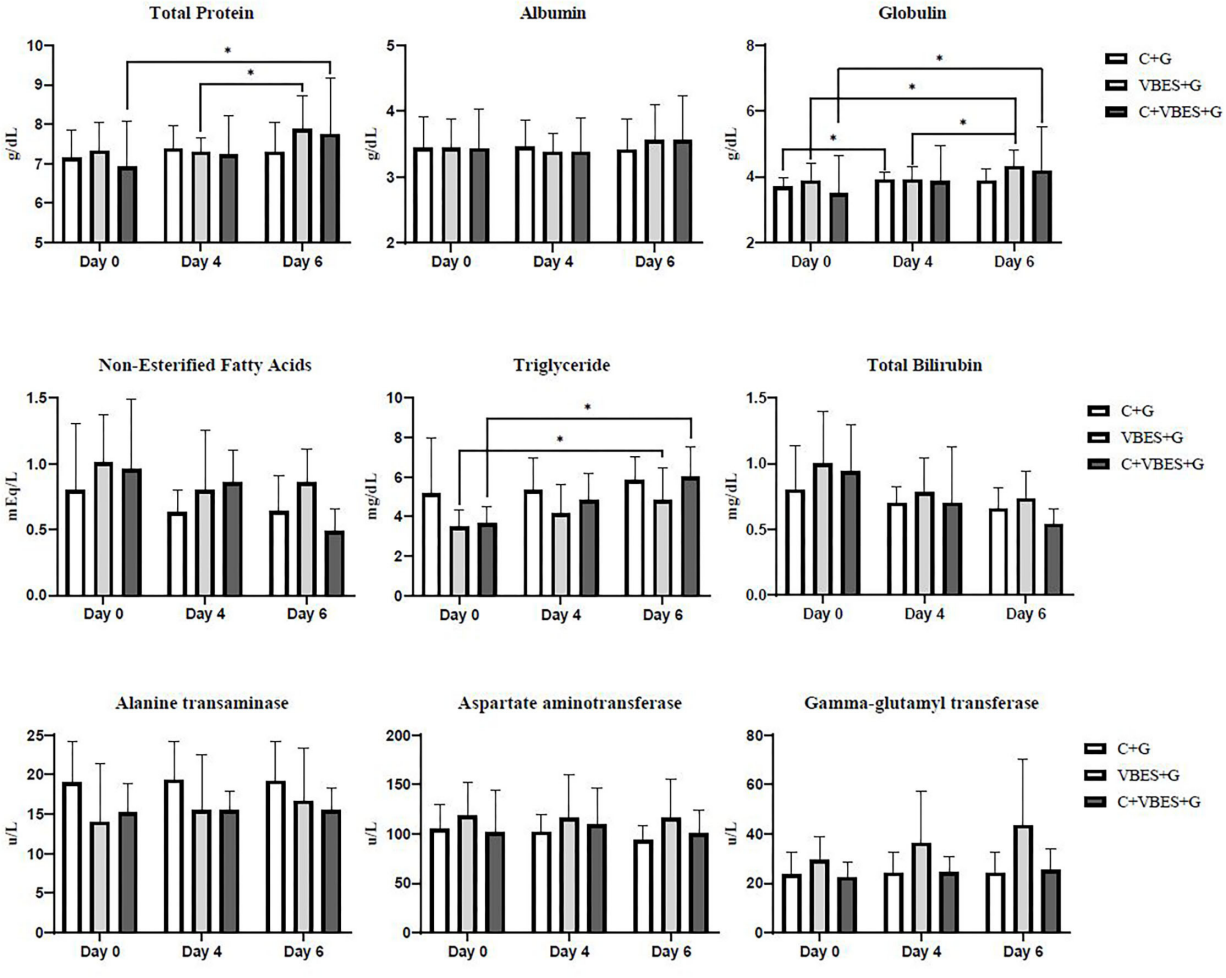
Total protein, albumin, globulin, non-esterified fatty acids, triglyceride, total bilirubin, alanine transaminase, aspartate aminotransferase, and gamma-glutamyl transferase on the pre-treatment day (Day 0), and on 1 day (Day 4) and 3 days (Day 6) after the last treatment day. C+G = levocarnitine and glycerin, VBES+G = vitamin B complex, vitamin E with selenium, and glycerin, C+VBES+G = levocarnitine, vitamin B complex, vitamin E with selenium, and glycerin. Data are presented as the mean ± standard deviation. ^*^p < 0.05.

The changes in the serum biochemical parameters were assessed. Most of serum biochemical values were not statistically significant. However, in the within-subjects contrast tests, the main effects of time were very significant in both TP and globulin concentrations (*p* ≤ 0.001). The interaction effect of group-by-time was significant in globulin (*p* < 0.05), but there was not significant difference of globulin between groups. Both VBES+G and C+VBES+G groups substantially increased the albumin concentration (0.12–0.18 g/dL) on day 6, compared with that in day 0. Globulin concentration significantly increased on day 6, compared with that on day 0 in both the VBES+G and C+VBES+G groups (0.43-0.7 g/dL). However, negligible changes by time in albumin (< 0.05 g/dL) and globulin (<0.22 g/dL) levels were observed in the C+G group. The C+VBES+G group showed a considerable decrease (0.10 mEq/L, day 4; 0.47 mEq/L, day 6) in NEFA, compared with that on day 0. Both C+G and VBES+G groups showed considerably decreased NEFA on day 4, but showed increased NEFA on day 6. All groups showed improvement in the TG and TB levels. The TG level improved significantly in both VBEST+G and C+VBES+G groups between day 0 and day 6 (*p* < 0.05). The C+VBES+G group showed the greatest improvement in the TG and TB. No significant changes in ALT, AST, and GGT levels were observed among groups (Table 5 and Figure 4).

**Table 5:** Interaction effect of time and group on total protein, albumin, globulin, and triglyceride concentration by repeated measures ANOVA.

**Source of variation**	**Sum of squares**	**Degrees of freedom**	**Mean squares**	***F***	***p*** **-value**
**Total protein**					
Time	2.434	2	1.217	8.222	0.001
Time*Group	1.144	4	0.286	1.933	0.131
Error	4.441	30	0.148		
Group	0.568	2	0.284	0.140	0.870
Error	30.341	15	2.023		
**Albumin**					
Time	0.105	2	0.052	1.260	0.298
Time*Group	0.114	4	0.029	0.686	0.607
Error	1.248	30	0.042		
Group	0.005	2	0.002	0.004	0.996
Error	9.921	15	0.661		
**Globulin**					
Time	1.736	2	0.868	19.513	<0.001
Time*Group	0.596	4	0.149	3.351	0.022
Error	1.334	30	0.044		
Group	0.494	2	0.247	0.146	0.865
Error	25.359	15	1.691		
**Triglyceride**					
Time	18.815	2	9.407	6.019	0.006
Time*Group	4.296	4	1.074	0.687	0.606
Error	46.889	30	1.563		
Group	14.704	2	7.352	1.738	0.209
Error	63.444	15	4.230		

## Discussion

This study was conducted to evaluate the effects of treatment with levocarnitine (C+G), vitamin B complex, and E with selenium (VBES+G), and the combination of levocarnitine and vitamin B complex and E with selenium (C+VBES+G), each in combination with glycerin, on the resolution of clinical ketosis during the postpartum transition period, based on blood BHBA, milk production, and serum biochemical parameters. The effect of increasing fatty acid influx into the liver, stimulating metabolism with reducing oxidative stress, and the synergy of both was examined in cows with clinical ketosis, which were fed a precursor for glucose synthesis. The cows treated with C+VBES+G tended to demonstrate an improvement in clinical ketosis along with improvements in BHBA, glucose, MY, serum proteins, NEFA, TG, and TB compared with those in cows in the other groups. This could help treat cows with ketosis and establish suitable prevention strategies.

Daily MY is speculated to be associated with the alleviation of clinical ketosis, especially serum glucose concentration. The C+VBES+G group showed the greatest increase in the amount of milk, improvements in the BHBA and serum glucose concentration. Both C+G and VBES+G increased MY and improved serum glucose concentration on day 4, compared with that on the onset day (day 0) of ketosis. However, both C+G and VBES+G decreased MY and glucose concentration on day 6, compared with that on day 4. This corresponds to the findings of previous studies, which showed that cows that overcame ketosis produced more milk than untreated cows (4, 12, 13, 38).

NEFA tended to be associated with the concentrations of BHBA and serum glucose, to greater or less degrees. The concentrations of BHBA and serum glucose improved as the NEFA concentration decreased. Stabilization of glucose concentration moderates NEFA mobilization, which reduces BHBA (1, 39). Here, the C+VBES+G group maintained the improvement in the BHBA, glucose, and NEFA concentrations without the treatment (from day 4 to 6), even though the other groups did not show an improvement in all of these parameters. This difference may derive from serum glucose concentration on day 4. In C+VBES+G, a high glucose concentration on day 4 might contribute to a decrease in NEFA mobilization and BHBA concentration. In contrast, C+G and VBES+G with low serum glucose concentration might fail to decrease NEFA mobilization and BHBA concentration.

Glycerin reduces NEFA and BHBA (22, 40, 41). In the present study, all groups showed decreased NEFA and BHBA levels. However, glycerin alone may be insufficient to overcome clinical ketosis. Three cases in the C+G group still showed clinical ketosis and two cases in the VBES+G group showed exacerbation to clinical ketosis during the time of no treatment. Glycerin may contribute to increased glucose concentrations, which was observed in all the groups. The rumen completely ferments glycerin to volatile fatty acids such as propionate and butyrate (42, 43). Propionate is converted to glucose via gluconeogenesis in the liver (44). Hepatic gluconeogenesis is responsible for over 90% of glucose production (45). Increased propionate is linked with increased glucose production.

Levocarnitine is involved in metabolic functions, including the transport of long chain free fatty acids from the cytosol to the mitochondrial matrix in the outer mitochondrial membrane. These fatty acids undergo β-oxidation to produce energy (46, 47). Fatty acid β-oxidation results in high concentrations of BHBA in the blood (48). Dietary levocarnitine supplementation increases the capacity to oxidize NEFA, which decreases fat accumulation in the liver (49). Improved fat accumulation and glucose output in the liver by carnitine supplementation ameliorate glucose status during early lactation (48). Furthermore, Owen et al. reported that the liver produces more glucose with carnitine supplementation, which increases metabolite flux through pyruvate carboxylase (50). In the present study, using levocarnitine alone with glycerin did not significantly improve the BHBA and glucose concentrations, and may thus be insufficient to improve ketosis. However, when the C+VBES+G group is compared with the VBES+G group, levocarnitine must considerably contribute to improvements in the BHBA and glucose concentrations and the persistence of its effect. The results indicate that fatty acids, which are transported into the mitochondrial matrix by levocarnitine, must be metabolized to ameliorate ketosis, and that levocarnitine substantially contributes to the resolution of ketosis once fatty acids can be metabolized properly in the liver. Holstein cows with clinical ketosis during the transition period may need materials to improve the influx of fatty acids into the mitochondria of the hepatic cell, as well as substances to improve the metabolism of hepatic cells.

Vitamin B complex and E with selenium may improve ketosis by supplementing the deficiencies in dairy cows with ketosis for efficient metabolism. Cows with ketosis show excessive lipid accumulation in the liver, which damages mitochondrial function, metabolism, and cellular signal transduction, and causes severe oxidative stress and apoptosis by weakening endogenous antioxidant defenses (10, 51, 52). The vitamin B complex plays essential roles in carbohydrate, protein, and fat metabolism, by serving as enzyme cofactors. Thiamine, riboflavin, and niacin contribute to converting pyruvate to acetyl-CoA and producing energy through the tricarboxylic acid (TCA) cycle (30, 53–57). Metabolic enzymes that require riboflavin also include glycerol-3-phosphate dehydrogenase, which is involved in glycerol phosphate shuttle and triglyceride synthesis in the liver (58). Nicotinamide adenine dinucleotide (NAD), which is converted from niacin, serves as a cofactor for β-hydroxybutyrate dehydrogenase to degrade ketone bodies (59). Pyridoxal phosphate, the biologically active form of pyridoxine, is involved in catalyzing transamination and catabolizing glycogen to glucose (60). Cyanocobalamin participates in the degradation of amino acids such as valine, isoleucine, methionine, and threonine, odd-chain fatty acids, and cholesterol to produce succinyl-CoA, an intermediate product of the TCA cycle (61). Vitamin E is low in cows with hepatic failure and functions as an antioxidant by preventing oxidative damage to hepatic tissue (62, 63). Selenoproteins, which selenium plays a major component of, function as essential enzymes to reduce oxidation and maintain homeostasis (64). Plasma selenium concentration is positively associated with the capacity of antioxidation, and the administration of selenium induces antioxidant enzymes in subclinical ketosis (10, 65). The accumulative effect of vitamin B complex and E with selenium resulted in recovery from clinical ketosis immediately after treatment, as well as improvement in the serum protein status. Malnutrition and liver atrophy result in insufficient protein synthesis, which is associated with hypoalbuminemia and hypoglobulinemia (66). Increased albumin, globulin, and TG may be linked with the improvement in liver function. This effect extended after 2 days without treatment. However, treatment without levocarnitine was less effective than that with levocarnitine in terms of concentrations of BHBA and glucose and improvement in milk yield. This is due to the role of levocarnitine. These results indicate that overcoming ketosis requires increased metabolism of fatty acids and BHBA through the TCA cycle and reduced oxidative stress, as well as reduced fatty acid concentrations through transportation from the cytosol to the mitochondria in the liver.

We made efforts to have cows of similarity in parity, age at calving, and incidence day of clinical ketosis in all groups, but we could not because these ketotic cases were natural, not induced. The C+VBES+G group, which had higher number of parity and age, showed better results. Based on which cows with more parity and older age are vulnerable to ketosis, C+VBES+G may be more effective than the other treatments. The influence of incidence day of clinical ketosis on the results are unclear. However, in this study, all cases were confined in the postpartum transition period (for 21days from the calving date) as mentioned in the materials and methods. The influence of incidence day of clinical ketosis might be limited.

To our knowledge, this is the first study indicating that clinical ketosis might be treated with levocarnitine and vitamin B complex and E with selenium along with oral administration of glycerin. Our findings suggest a new treatment in which dairy Holstein cows may overcome clinical ketosis using this combination. However, further studies are needed to confirm and clarify their effects on various cows. The administration method and dosage of levocarnitine, vitamin B complex, vitamin E, selenium and glycerin should be studied further because we just followed the instructions of pharmaceutical companies and a previous study (31).

In conclusion, three types of treatment using levocarnitine and vitamin B complex and E with selenium, in combination of glycerin, were applied to cows with clinical ketosis. Using all these treatments in combination resulted in improvements in clinical ketosis compared with that achieved with each treatment. The synergistic effect of glycerin, levocarnitine, and vitamin B complex and E with selenium is possible to help cows to overcome clinical ketosis with improved BHBA, glucose, MY, serum protein, and NEFA. In addition, this effect might persist after stopping the treatment. Our study thus suggests a new treatment strategy for ketosis. Further studies are required to elucidate the effect of these treatments for application to various cows.

## Data Availability Statement

The raw data supporting the conclusions of this article will be made available by the authors, without undue reservation.

## Ethics Statement

The animal study was reviewed and approved by the Institutional Animal Care and Use Committee (IACUC) at the National Institute of Animal Science, Republic of Korea (approved number: NIAS-2020127).

## Author Contributions

SH: conceptualization, methodology, software, validation, investigation, data curation, writing original draft preparation, visualization, and project administration. SK and MH: project administration and funding acquisition. JL, HC, and DK: methodology. JP: conceptualization, validation, data curation, writing review and editing, and supervision. All authors contributed to the article and approved the submitted version.

## Funding

This research was funded by The improvement of animal diseases control in National Institute of Animal Science (Project No. PJ01567603) project at National Institute of Animal Science, Rural Development Administration, Republic of Korea.

## Conflict of Interest

The authors declare that the research was conducted in the absence of any commercial or financial relationships that could be construed as a potential conflict of interest.

## Publisher's Note

All claims expressed in this article are solely those of the authors and do not necessarily represent those of their affiliated organizations, or those of the publisher, the editors and the reviewers. Any product that may be evaluated in this article, or claim that may be made by its manufacturer, is not guaranteed or endorsed by the publisher.
